# B-cell activating factor and IL-21 levels predict treatment response in autoimmune hepatitis

**DOI:** 10.1016/j.jhepr.2022.100460

**Published:** 2022-02-22

**Authors:** Maaike Biewenga, Sebastiaan Heidt, Manon Vergunst, Camiel M.J. Marijnissen, Rob A. de Man, Annemiek A. van der Eijk, Adriaan J. van der Meer, Leendert A. Trouw, Bart van Hoek

**Affiliations:** 1 Department of Gastroenterology and Hepatology, Leiden University Medical Center, Leiden, The Netherlands; 2 Department of Immunology, Leiden University Medical Center, Leiden, The Netherlands; 3 Department of Gastroenterology and Hepatology, Erasmus Medical Center, Rotterdam, The Netherlands; 4 Department of Viroscience, Erasmus Medical Center, Rotterdam, The Netherlands

**Keywords:** Autoimmune hepatitis, BAFF, IL-21, B-cell immunity, Biochemical remission, AIH, autoimmune hepatitis, ALT, alanine aminotransferase, AP, alkaline phosphatase, AST, aspartate aminotransferase, BAFF, B-cell activating factor of the tumour necrosis family, GGT, gamma-glutamyltransferase, LKM-1, liver–kidney–microsomal type 1, MRCP, magnetic resonance cholangiopancreaticography, PBC, primary biliary cholangitis, PBMC, peripheral blood mononuclear cell, PSC, primary sclerosing cholangitis, UDCA, ursodeoxycholic acid, ULN, upper limit of normal

## Abstract

**Background & Aims:**

Increased serum IgG and autoantibodies suggest involvement of B cells in autoimmune hepatitis (AIH). The aim of this study was to assess levels of B cell activating factor of the tumour necrosis family (BAFF), IL-21, and circulating B cell populations in AIH and correlate these to treatment response.

**Methods:**

BAFF and IL-21 levels were determined in 66 patients with AIH before treatment and 10 healthy controls. Flow cytometry was performed on circulating B cells of 10 patients with AIH and 12 healthy controls.

**Results:**

Based on BAFF and IL-21 levels, untreated patients with AIH were divided into 3 groups: 27 (41%) patients with normal BAFF and IL-21 (normal BAFF), 27 (41%) patients with elevated BAFF but normal IL-21 (high BAFF), and 12 (18%) patients with elevated IL-21 (high IL-21). The high BAFF group presented with higher bilirubin compared with the normal BAFF and high IL-21 groups (159 *vs*. 26 *vs*. 89 μmol/L; *p* = 0.001; Mann–Whitney *U* test). After 12 months of treatment, 54% of the high BAFF group reached remission compared with 34% of the normal BAFF group and 0% of the high IL-21 group (*p* = 0.006, Chi-square test). During follow-up, 3 patients (25%) with high IL-21 developed primary sclerosing cholangitis (PSC) variant syndrome. Autoimmune-associated B cells were increased in patients with AIH compared with healthy controls (4.4 *vs*. 1.4%; *p* = 0.003, Mann–Whitney *U* test). BAFF levels were correlated positively with naïve B cells (*p* = 0.01) and negatively with class-switched B cells (*p* = 0.003) and nonclass-switched B cells (*p* = 0.005, Spearman correlation).

**Discussion:**

Using BAFF and IL-21, we identified different immunological phenotypes of AIH with a different presentation, treatment response, and outcome. Patients with high IL-21 had the poorest treatment response and a risk of developing PSC variant syndrome. BAFF level was related to shifts in circulating B-cell populations.

**Lay summary:**

In patients with untreated autoimmune hepatitis (AIH), circulating B-cell activating factor of the tumour necrosis family (BAFF), IL-21, and B-cell populations were determined. Three subgroups were identified: with (1) normal BAFF and IL-21, (2) elevated BAFF and normal IL-21, and (3) elevated IL-21. Remission after 1-year treatment occurred in 54, 34, and 0% in Groups 1, 2, and 3, respectively. Group 2 had higher bilirubin, indicating more liver dysfunction. In 25% of patients with high IL-21, AIH-PSC variant syndrome developed, but none in the other groups. Autoimmune-associated B cells were elevated and BAFF levels correlated with certain B cells.

## Highlights

• In AIH, 3 subgroups were identified: with (1) both normal BAFF and IL-21, (2) elevated BAFF but normal IL-21, and (3) elevated IL-21.
• Remission after 1-year treatment occurred in 54, 34, and 0% in groups 1, 2, and 3, respectively.
• Group 2 had higher bilirubin, indicating more severe liver dysfunction.
• AIH-PSC variant syndrome developed in 25% of patients with high IL-21, but not in the other groups.
• Autoimmune-associated B cells were elevated, and BAFF levels correlated with certain B cells.

## Graphical abstract

**Figure:**
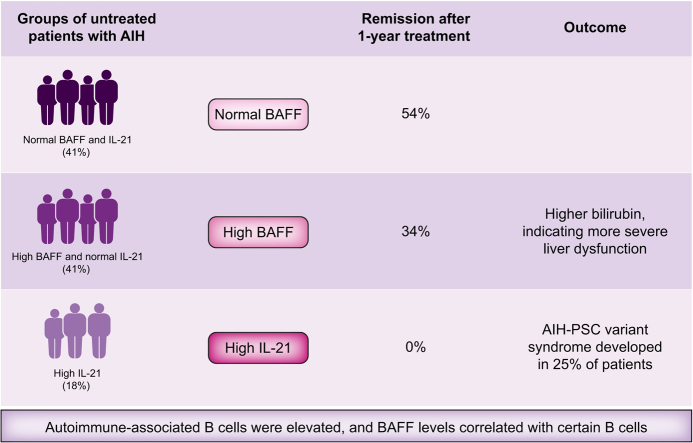

## Introduction

First-line treatment of autoimmune hepatitis (AIH), a chronic autoimmune disease of the liver, consists of glucocorticoids combined with azathioprine. The aim of treatment is to reach and maintain biochemical remission to prevent progression to liver cirrhosis, liver transplantation, or death. Complete biochemical remission is defined as normal serum aminotransferases and immunoglobulin G (IgG). In a considerable group of patients only a partial biochemical remission is reached.^1^ Partial biochemical remission is a risk factor for disease progression.^2^ In 10% of the patients with AIH, signs of primary biliary cholangitis (PBC) or primary sclerosing cholangitis (PSC) are present, called AIH variant syndromes.^1^^,^^3^

Current practice guidelines advise a uniform induction treatment of prednisolone and azathioprine in all patients with AIH.^1^^,^^4^ However, early identification of difficult-to-treat patients using biomarkers could lead to more personalised treatment.

Autoantibodies and elevated total IgG in serum, as well as the pronounced presence of plasma cells in the liver biopsy, are prominent features of AIH, which all relate to B-cell immunity.^5^ B cells are the cornerstone of humoral immunity. Immature B cells can develop through transitional B cells to naïve B cells. After activation, naïve B cells will differentiate to (non)class-switched memory B cells or plasma cells.^6^ Autoimmunity-associated B cells belong to a specific subgroup of B cells frequently found in patients with chronic inflammation.^7^ B-cell activating factor of the tumour necrosis family (BAFF), a B-cell cytokine, is essential for the survival of transitional and naïve B cells.^8^ Elevated BAFF can lead to survival of autoreactive B cells. In mice, this leads to increased serum Ig levels, antinuclear antibodies, and B-cell hyperplasia.^9^ A previous study showed that BAFF levels were elevated in patients with AIH at diagnosis compared with healthy controls.^10^

IL-21 can stimulate differentiation of B cells towards plasma cells and is produced by follicular helper T cells. Elevated IL-21 levels have been reported previously in untreated patients with AIH.^11^^,^^12^

The aim of this study was to assess both BAFF and IL-21 levels in patients with AIH before and during treatment. The secondary aim was to identify and compare immunological subgroups based on IL-21 and BAFF levels with respect to presentation, treatment response, and long-term outcome. The tertiary aim was to assess circulating B-cell populations in AIH and relate these to BAFF and IL-21 levels.

## Patients and methods

In this retrospective study, all patients with AIH with available serum samples before treatment between 1996 and 2020 were eligible for inclusion. The revised International Auto Immune Hepatitis Group criteria were used for the diagnosis of AIH, and patients with a pretreatment score of 10 or higher – compatible with definite or probable AIH – were included.^13^ Serum samples were obtained from the Leiden University Medical Center and Erasmus Medical Center biobanks. The first available serum sample after at least 6 months of immunosuppressive treatment was also included. In addition to those of patients with AIH, serum samples of 10 healthy controls with similar age and sex were included. The study protocol was approved *a priori* by the Leiden medical ethical committee (B19.001 and B20.013), and informed consent was obtained. The study complied with the latest version of the Declaration of Helsinki.

All clinical data were retrieved from electronic patient files with permission. Definitions for treatment response were according to the EASL guidelines: biochemical remission was defined as normalisation of aminotransferases and IgG, and partial response as improvement of aminotransferases and IgG without normalisation.^1^

### ELISA

BAFF and IL-21 levels were determined using DuoSet ELISA (R&D Systems, Minneapolis, MN, USA) according to the manufacturer’s protocol. If BAFF or IL-21 was measured to be below the lower limit of detection or above the upper limit of detection, the lower limit or upper limit of detection was used as the value of BAFF and/or IL-21 of this sample in further analysis. Based on the BAFF and IL-21 levels in healthy controls, cut-off levels for elevated BAFF and IL-21 were determined. Cut-off levels were determined before any clinical association analysis was done. Patients were divided into subgroups based on BAFF and IL-21 levels and compared regarding levels of bilirubin, aspartate aminotransferase (AST), alanine aminotransferase (ALT), alkaline phosphatase (AP), gamma-glutamyltransferase (GGT), and IgG at diagnosis and after 12 months of treatment, proportion of biochemical remission, development of variant syndrome, and outcome regarding liver transplantation or liver-related death.

### Flow cytometry

Peripheral blood mononuclear cells (PBMCs) were isolated from 10 consecutive patients with AIH before treatment and 12 healthy controls by Ficoll-Paque gradient centrifugation and stored in liquid nitrogen in the Leiden University Medical Center biobank until further use between 2018 and 2020. After thawing the PBMCs using benzonase, flow cytometry using an Aurora-3L flow cytometer was performed according standard protocols using 7AAD and the following antibodies: CD45-KrO (J33), CD38-APC-A750 (LS198-4-3), CD19-ECD (J3-119), CD21-PE (BL13), CD24-APC (ALB9), CD27-PC7 (1A4CD27), IgD-FITC (IA6-2), and IgM-PB (SA-DA4; all from Beckman Coulter, Brea, CA, USA).

Using these markers, the following B-cell populations were identified: total B cells, naïve B cells, transitional B cells, nonclass-switched B cells, class-switched B cells, autoimmunity-associated B cells, and subsets according to the Bm1–Bm5 classification.^14^ The definitions of these cell populations are shown in Table 1, and the gating strategy is detailed in Fig. S1.

**Table 1:** **Definitions of B-cell populations**.

B-cell population (functional description)	Markers
B cells	CD45+ CD19+
Transitional B cells	CD45+ CD19+ CD27- IgD+ CD38+ CD24+
Naïve B cells	CD45+ CD19+ CD27- IgD+ CD38-
Non-class-switched memory B cells	CD45+ CD19+ CD27- IgD+ CD38- IgM+
Class-switched memory B cells	CD45+ CD19+ CD27+ IgD- CD38- IgM-
Autoimmunity-associated B cells	CD45+ CD19+ CD21- CD38-
Bm 1 (naïve B cells)	CD45+ CD19+ IgD+ CD38-
Bm 2 (activated naïve B cells)	CD45+ CD19+ IgD+ CD38+
Bm 2′ + Bm 3δ 4δ (germinal centre founder cells)	CD45+ CD19+ IgD+ CD38++
Early Bm 5 (early memory B cells)	CD45+ CD19+ IgD- CD38+
Bm 5 (late memory B cells)	CD45+ CD19+ IgD- CD38-

### Statistics

Statistical analysis was performed with IBM SPSS version 25 (SPSS, Chicago, IL, USA). Data are presented as median (range) unless indicated otherwise. Clinical variables were corrected for the upper limit of normal (ULN) as different ULNs were used in the participating medical centres. The Mann–Whitney *U* test was used for nonpaired continuous variables, the Wilcoxon signed-rank test was used for paired continuous variables, and the Chi-square test was used for categorical variables. To assess correlation between continuous variables in normally distributed data, linear regression was used, and in not normally distributed data, Spearman correlation was used. Kaplan–Meier survival analysis with the log-rank test was used to assess the time to remission. A value of *p* <0.05 was considered significant. Calculated sample size with the 2-proportions test (ClinCalc, Chicago, IL, USA) with alpha 5% and beta 80% for remission in 54 *vs.* 0% was 18 (2×9), and that with alpha 10% and beta 70% for 31% *vs*. 0% was 24 (2×12), whereas it was 86 (2×43) for 31 *vs*. 54%, and 14 (2×7) with alpha 5% and beta 80% for BAFF level 500±100 *vs*. 350, and 2 (2×1) for IL-21 level 10 *vs*. 400±100.

## Results

In total, 66 patients with AIH were included with 66 serum samples before treatment and 26 serum samples during treatment. Of the 66 samples before treatment, 62 were taken at diagnosis and 4 were taken at the time of a relapse after immunosuppressive therapy was completely stopped and before it was restarted. Liver biopsy was performed at diagnosis in 62 patients (94%), and no signs of variant syndromes were present. In 62 patients, induction treatment with prednisolone (n = 54) or budesonide (n = 8) was started. No treatment was started in 3 patients: none in 2 because of mild disease and 1 patient refused treatment. For the 26 patients with a sample during treatment, the median time between samples was 55 months (range 6–180 months), and treatment at the second sample consisted of prednisolone in 18 patients (72%; median dose 10 mg [range 2.5–25 mg]), thiopurines in 14 patients (54%), mycophenolate mofetil in 9 patients (35%), cyclosporine in 1 patient (4%), tacrolimus in 1 patient (4%), and ursodeoxycholic acid (UDCA) in 1 patient (4%). Cirrhosis was present in 25 patients (38%), and the median model for end-stage liver disease score was 16.5 (range 6–29). In addition, 10 samples of healthy controls were included. Age and sex were comparable between healthy controls (median 40.5 years [range 24–69]; 70% female) and patients (median age 49.5 years [range 6–75]; 72% female). Median follow-up was 95 months (range 1–412 months).

### BAFF levels before and during treatment

**Fig. 1:**
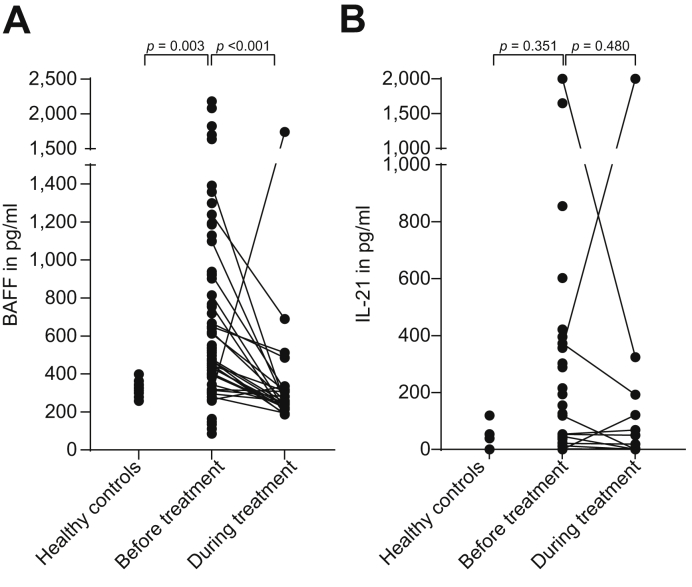
Levels of BAFF and IL-21 in patients with AIH and healthy controls. (A) Compared with healthy controls, BAFF levels were higher before treatment (*p* = 0.003, Mann–Whitney *U* test), and compared with pre-treatment levels, they decreased after treatment (*p* <0.001, Wilcoxon signed-rank test). (B) IL-21 was elevated only in a subpopulation of patients, and with treatment, these levels remained unchanged compared with pre-treatment values (*p* = 0.480, Wilcoxon signed-rank test). AIH, autoimmune hepatitis; BAFF, B-cell activating factor of the tumour necrosis family.

Despite a wide range, the median serum BAFF level in patients with AIH before treatment was significantly higher compared with healthy controls (517 pg/ml [range 85–2182] *vs*. 326 pg/ml [range 258–398]; *p* = 0.003). Upon treatment, the median BAFF level decreased to 251 pg/ml (range 186–1740; *p* <0.001; Fig. 1). In 6 patients (9%) BAFF levels were <200 pg/ml (range 85–163) before treatment, which was lower than measured in any of the healthy controls.

### IL-21 levels before and during treatment

Concentrations of IL-21 were also determined in all samples. In 7 serum samples (70%) of healthy controls, 38 samples (58%) before treatment, and 20 samples (71%) during treatment, IL-21 was lower than 10 pg/ml, the lower limit of detection. In 3 patients, IL-21 was higher than 2,000 pg/ml, the upper limit of detection. IL-21 levels were not significantly different before and during treatment (Fig. 1; *p* = 0.480).

BAFF and IL-21 levels were correlated with AST, ALT, and IgG levels. BAFF and IL-21 were significantly (*p* = 0.016) but very weakly correlated with each other (*R*^2^ = 0.076). AST and ALT were also weakly correlated with BAFF with a *R*^2^ of 0.206 and 0.191, respectively (*p* <0.001 for both). IL-21 was not correlated with AST and ALT (*p* = 0.989 and *p* = 0.334, respectively). IgG was weakly correlated with IL-21 (*R*^2^ = 0.096; *p* = 0.017) but not with BAFF (*R*^2^ = 0.006; *p* = 0.564; Fig. S2).

### Comparison of the normal BAFF, high BAFF, and high IL-21 subgroups

Because a wide range of BAFF and IL-21 levels was found in patients with AIH, subgroups based on BAFF and IL-21 levels were defined and compared regarding presentation, treatment response, and long-term outcome. Based on BAFF and IL-21 levels in healthy controls, BAFF level >500 pg/ml and IL-21 >200 pg/ml were used as cut-off values to create subgroups. Patients were divided into 3 subgroups: 27 (41%) patients with BAFF <500 pg/ml and IL-21 levels <200 pg/ml (normal BAFF group), 27 (41%) patients with BAFF >500 pg/ml but IL-21 levels <200 pg/ml (high BAFF group), and 12 (18%) patients with IL-21 levels >200 pg/ml (high IL-21 group). As only 12 patients had high IL-21 levels, this group was not divided based on BAFF levels. A BAFF level of >500 pg/ml was present in 7 of 12 patients (58%) with high IL-21.

The median BAFF level was 344 pg/ml (range 85–496) in the normal BAFF group, 814 pg/ml (range 511–1,825) in the high BAFF group, and 600 pg/ml (range 112–2,182) in the high IL-21 group. The median IL-21 was <10 (range <10–58) in the normal BAFF group, <10 (range <10–193) in the high BAFF group, and 408 (range 215 to >2,000) in the high IL-21 group.

Presentation of AIH was significantly different between the subgroups: patients with high BAFF had a higher median bilirubin compared with patients with high IL-21 and patients with normal BAFF (159 μmol/L *vs*. 89 μmol/L *vs*. 26 μmol/L; *p* = 0.001). ALT and AST were also higher in patients with high BAFF (19.8×ULN [range 0.7–102] for ALT; 22×ULN [range 1.5–142] for AST) compared with patients with high IL-21 (6.8×ULN [range 0.9–40] for ALT; 6.7×ULN [range 1.6–67] for AST) and patients with normal BAFF (6.1×ULN [range 0.9–29], *p* = 0.001, for ALT; 6.0×ULN [range 1.2–44.5], *p* = 0.002, for AST; Table 2). In both included patients with AIH type 2, characterised by anti-liver–kidney–microsomal type 1 (anti-LKM-1) antibodies, IL-21 was high.

**Table 2:** **Characteristics of immunological phenotypes based on BAFF and IL-21 before and during treatment**.

	Normal BAFF∗	High BAFF†	High IL-21‡	*p* value
Patients (N)	27 (41%)	27 (41%)	12 (18%)	
Sex	18 (67%)	21 (78%)	9 (75%)	0.645
Age (years)	51 (12–74)	54 (12–74)	44 (6–75)	0.507
Before treatment				
Bilirubin (μmol/L)	26 (7–286)	159 (10–773)	89 (13–405)	0.001
AST×ULN	6.0 (1.2–44.5)	22.0 (1.5–142)	6.7 (1.6–67.7)	0.002
ALT×ULN	6.1 (0.9–29.0)	19.8 (0.7–102)	6.8 (0.9–40.0)	0.001
AP×ULN	1.5 (0.7–19.1)	1.7 (0.8–3.9)	2.6 (0.97–12.6)	0.062
GGT×ULN	4.8 (0.4–15.0)	5.1 (0.8–19.2)	6.1 (1.2–12.7)	0.45
Albumin (g/L)	40 (20–45)	37.5 (23–49)	33 (21–42)	0.051
Platelets	199 (48–362)	235 (84–339)	174 (43–481)	0.512
IgG (g/L)	20.1 (12–54)	26.3 (8.6–41)	32.1 (10–60.7)	0.194
ANA	17 (65%)	20 (74%)	7 (58%)	0.593
Anti-SMA	14 (54%)	15 (58%)	7 (58%)	0.949
Anti-LKM-1	0 (0%)	0 (0%)	2 (17%)	0.010
AMA	0 (0%)	2 (8%)	0 (0%)	0.221
Cirrhosis	9 (33%)	9 (33%)	7 (58%)	0.271
Treatment				
Prednisolone	19 (73%)	25 (93%)	10 (83%)	0.166
Budesonide	5 (19%)	2 (7%)	1 (8%)	0.381
Thiopurines	21 (81%)	25 (93%)	9 (75%)	0.043
12 months				
AST×ULN	1.64 (0.49–4.74)	0.87 (0.48–38)	1.51 (0.87–3.03)	0.002
ALT×ULN	1.24 (0.33–7.79)	0.76 (0.29–9.93)	1.51 (0.41–2.56)	0.032
AP×ULN	0.91 (0.44–2.04)	0.75 (0.38–9.26)	1.49 (0.67–3.44)	0.019
GGT×ULN	1.63 (0.34–8.58)	1.05 (0.26–5.32)	3.3 (0.76–10.33)	0.009
IgG (g/L)	12.9 (7.65–32)	12.5 (7.5–21.7)	15.5 (10.8–27.8)	0.294
First remission within 12 months	8 (31%)	13 (54%)	0 (0%)	0.006
Long-term outcome				
Follow-up	95 (1–412)	94 (1–289)	95 (1–252)	
Development PSC	0 (0%)	0 (0%)	3 (25%)	0.001
Liver transplantation	0 (0%)	0 (0%)	1 (8%)	0.102
Liver-related death	0 (0%)	3 (11%)	1 (8%)	0.216

*p* <0.050 was the level of significance, Mann–Whitney *U* test for continuous variables and Chi-square for categorical variables. ALT alanine aminotransferase; AMA, antimitochondrial antibodies; ANA, antinuclear antibodies; AP alkaline phosphatase; AST, aspartate aminotransferase; BAFF, B-cell activating factor of the tumour necrosis family; GGT gamma-glutamyltransferase; LKM-1, liver–kidney–microsomal type 1; PSC primary sclerosing cholangitis; SMA, smooth muscle antigen; ULN, upper limit of normal.

∗ Patients with BAFF <500 pg/ml and IL-21 <200 pg/ml before treatment.

† Patients with BAFF >500 pg/ml and IL-21 <200 pg/ml before treatment.

‡ Patients with IL-21 >200 pg/ml before treatment.

Response to treatment differed between the subgroups: in patients with high BAFF, in whom median AST and ALT were the highest at diagnosis, median AST and ALT at 12 months of treatment were lower compared with the patients with normal BAFF and the patients with high IL-21 (*p* = 0.002 and *p* = 0.032, respectively). In patients with high IL-21, the cholestatic parameters AP and GGT at 12 months were significantly higher compared with patients with high BAFF and normal BAFF (1.49 *vs*. 0.75 *vs*. 0.91×ULN for AP, *p* = 0.019; 3.3 *vs*. 1.05 *vs*. 1.63×ULN for GGT, *p* = 0.009). First complete biochemical remission was reached within 12 months in 54% of the patients with high BAFF and 31% of the patients with normal BAFF. No patients with high IL-21 reached remission within 12 months (*p* = 0.006; Table 2).

**Fig. 2:**
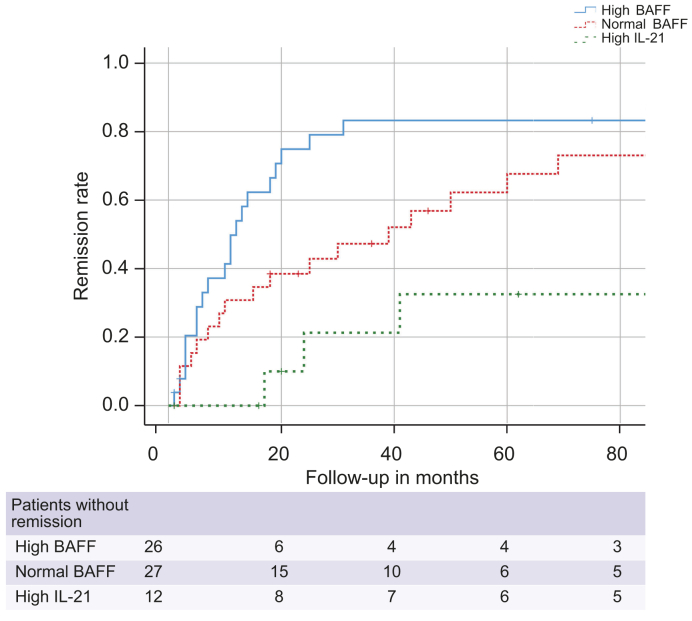
Treatment response in BAFF- and IL-21-based subgroups (Kaplan–Meier survival analysis). Time to first biochemical remission during follow-up was significantly longer in patients with high IL-21 than in patients with high BAFF (*p* = 0.001) and in patients with normal BAFF (*p* = 0.045). Patients with high BAFF tended to reach remission faster than patients with normal BAFF (*p* = 0.052). A value of *p* <0.050 was the level of significance (log-rank test). BAFF, B-cell activating factor of the tumour necrosis family.

Kaplan–Meier survival analysis was used to assess time to remission during follow-up (Fig. 2). During the entire follow-up, 17 patients with normal BAFF, 21 patients with high BAFF, and only 3 patients with high IL-21 reached complete biochemical remission. Compared with patients with high IL-21, patients with high BAFF and patients with normal BAFF reached remission more often (*p* = 0.001 for high BAFF; *p* = 0.045 for normal BAFF). The median time to remission tended to be shorter in patients with high BAFF compared with normal BAFF (12 *vs*. 39 months) although the difference was just not significant (*p* = 0.052).

Lastly, the long-term outcome including development of variant syndromes and occurrence of liver transplantation and liver-related death was compared between the subgroups. In the normal BAFF group, no patients developed variant syndrome, received a liver transplantation, or died from liver-related causes. In the high BAFF group, 3 patients died of liver-related causes after 2, 75, and 247 months, whereas no patients received a liver transplantation or developed variant syndrome. In the high IL-21 group, 3 patients (25%) were diagnosed with PSC variant syndrome after 11, 19, and 72 months based on features of PSC on magnetic resonance cholangiopancreaticography (MRCP) (*p* = 0.001). One patient with high IL-21 received a liver transplantation after 1 month as a result of treatment failure, and 1 patient died of a liver-related cause after 117 months.

### B-cell subpopulations and BAFF

**Fig. 3:**
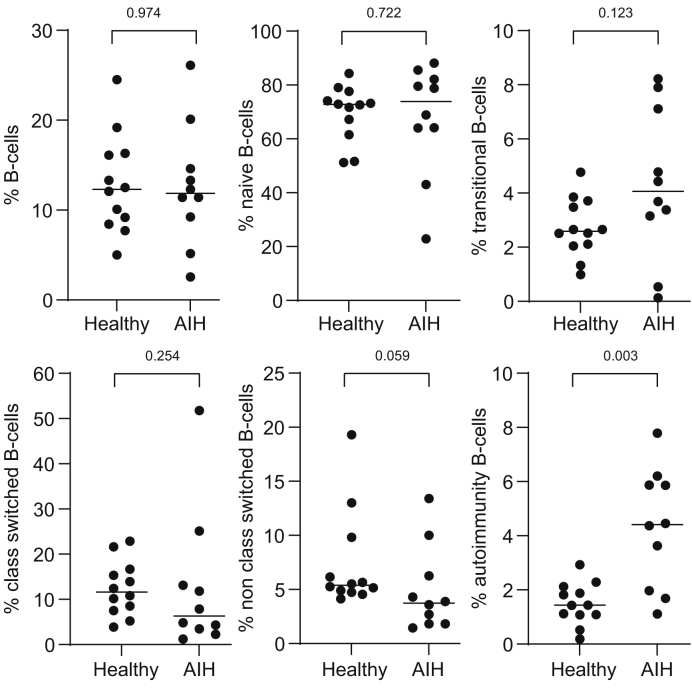
Prevalence of different B-cell populations in patients with AIH and healthy controls. All B-cell populations are expressed as percentage of B cells except for the total of B cells, which is expressed as percentage of lymphocytes. Median percentages are indicated by lines. The number on top of each figure indicates the *p* value, with *p* <0.050 as the level of significance (Mann–Whitney *U* test). AIH, autoimmune hepatitis.

**Fig. 4:**
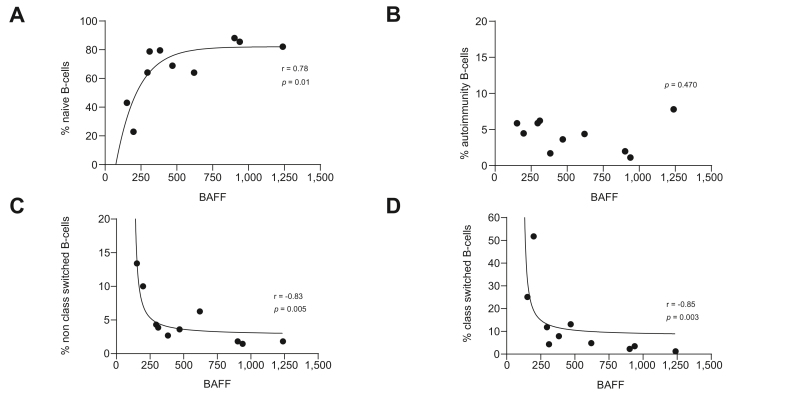
Correlation between BAFF level and B-cell populations. (A) Naïve B cells (Spearman correlation *r* = 0.78; *p* = 0.01), (B) autoimmunity-associated B cells (Spearman correlation *r* = -0.26; *p* = 0.470), (C) no-nclass-switched B cells (Spearman correlation *r* = -0.83; *p* = 0.005), and (D) class-switched B cells (Spearman correlation *r* = -0.85; *p* = 0.003). A *p* value <0.050 was considered significant. BAFF, B-cell activating factor of the tumour necrosis family.

B-cell subpopulations in patients with AIH before treatment were determined by flow cytometry and compared with healthy controls and correlated with BAFF levels. Autoimmunity-associated B cells were more prevalent in patients with AIH compared with healthy controls (median 4.4 *vs*. 1.4%, *p* = 0.003). All other subpopulations were not significantly different between patients with AIH and healthy controls, although a trend towards more transitional B cells and less nonclass-switched B cells in AIH was present (*p* = 0.123 and *p* = 0.059, respectively; Fig. 3 and Fig. S3). Spearman’s correlation of B-cell populations and BAFF showed that an increase in BAFF was related to an increase in naïve B cells (*p* = 0.01; *r* = 0.78) and to a decrease in class-switched B cells (*p* = 0.003; *r* = -0.85) and nonclass-switched B cells (*p* = 0.005; *r* = -0.83; Fig. 4). The prevalence of autoimmunity-associated B cells was not correlated with BAFF (*p* = 0.470). As only 1 of these 10 patients had an elevated IL-21, this analysis was not performed for IL-21.

## Discussion

Elevated IgG, autoantibodies, and presence of plasma cells in the liver biopsy suggest an important role for B-cell immunity in AIH. This study showed that patients with AIH can be divided into 3 immunological phenotypes based on the B-cell directed cytokines BAFF and IL-21 levels, with differences in presentation, treatment response, and long-term outcome. This may provide possibilities for a more personalised treatment in the future. To the best of our knowledge, this is the first study combining both markers and correlating these markers to treatment response, long-term outcome, and circulating B-cell subpopulations.

BAFF is mainly produced by dendritic cells, monocytes, and macrophages and is necessary for B-cell survival. Elevated BAFF levels can lead to increased survival of autoreactive B cells and IgG class switch in these cells.^9^ Serum BAFF was reported to be elevated in AIH and correlated with serum aminotransferases and IgG.^10^^,^^15^ IL-21, which is produced by Th17 T cells and follicular helper T cells in the germinal centre, can also stimulate B cells to perform class switch towards IgG and stimulates the development into plasma cells.^16^ Another function of IL-21 is to inhibit the function of regulatory T cells in 2 ways. Firstly, IL-21 enhances the ability of T cells to resist the suppression of regulatory T cells. Secondly, IL-21 reduces the production of IL-2 in all T cells, but IL-21 substitutes IL-2 as a growth factor in all T cell populations except for regulatory T cells, leading to a decrease in the absolute number of regulatory T cells.^17^ Reports on the functional and numerical impairment of regulatory T cells in AIH are currently conflicting.^18^^,^^19^ Two studies described an increased serum IL-21 in patients with AIH before treatment.^11^^,^^12^

In the current study, patients with AIH were divided into subgroups based on BAFF and IL-21 levels. Patients with high BAFF levels without elevated IL-21 presented often with high aminotransferases and jaundice. This correlation was also shown by a previous study.^10^ Patients with high BAFF had the best treatment response with the highest remission rate and the lowest level of aminotransferases at 12 months. Previously high aminotransferases at diagnosis were reported to be correlated with a better long-term survival.^20^ Fast decrease of aminotransferases during treatment and complete biochemical remission at 12 months were also associated with a better long-term survival.^21^ In this study, the number of patients was too small to further correlate BAFF with long-term survival. However, based on the treatment response, patients with high BAFF at diagnosis would likely have the best long-term outcome.

Patients with high IL-21 levels tended to present with a more cholestatic profile compared with patients with high BAFF and patients with normal BAFF. Especially after 12 months of treatment, AP and GGT were significantly higher compared with patients with high BAFF and normal BAFF. Three patients with high IL-21 developed AIH-PSC variant syndrome during follow-up compared with none of the patients with normal IL-21. Polymorphisms in the IL-21 gene have been associated with an increased risk for development of PSC^22^^,^^23^ but not with an increased risk for AIH type 1.^24^ In addition to a higher risk for development of AIH-PSC variant syndrome, patients with high IL-21 had the poorest response to treatment, as no patients with high IL-21 reached remission within 12 months.

In 40% of the patients, BAFF and IL-21 were not elevated compared with healthy controls. These patients tended to present with lower aminotransferases and less jaundice compared with patients with high BAFF or high IL-21. In some patients, BAFF levels were even lower than in healthy controls. It is possible that other immunological mechanisms play a role in these patients. A limitation of this study was that the cut-off values to discriminate these groups were based on a small group of healthy controls. It is possible that better cut-off values exist to discriminate these phenotypes. The number of included patients was too small to analyse the difference between treatment regimes.

In line with a previous article on immune cell populations in AIH, no difference was found in the composition of major B-cell populations of patients with AIH compared with healthy controls.^25^ It is possible that, owing to a limited number of samples and the use of frozen material, more delicate changes may have been missed and changes in absolute cell numbers could not be addressed. Plasma cells were not analysed in this study as the vast majority reside in the bone marrow, which we could not sample in this study, whereas the percentage of circulating plasma cells could not be determined because of the use of frozen PBMCs. An increase in BAFF led to a shift to more naïve B cells and less nonclass-switched and class-switched memory B cells. BAFF stimulates survival of especially transitional and naïve B cells, whereas memory cells are not dependent on BAFF, which explains this shift.^8^ This shift shows that, most likely, BAFF is not only a biomarker but also an active cytokine influencing the circulating B cells in patients with AIH. However, this should be further investigated in trials where BAFF inhibition is tested. These shifts in B-cell immunity might explain differences in presentation and treatment response, but more research into this important question is necessary.

Autoimmunity-associated B cells were increased in patients with AIH. This subset is expanded in patients with chronic infection and autoimmune diseases.^7^ Autoimmunity-associated B cells express BAFF-receptor but do not rely on BAFF for survival. IL-21 has been described to stimulate formation of autoimmunity-associated B cells.^26^ More research is needed to fully elucidate the role of these cells in AIH and autoimmune disease in general.

Surprisingly, BAFF and IL-21 levels were not or only weakly correlated with the total IgG level. In contrast to BAFF and IL-21, IgG level at diagnosis was not correlated with treatment response or long-term outcome.^27^ As IgG in AIH does not or minimally activate the complement system, hepatic injury in AIH does not seem to be caused by autoantibodies.^28^

The identification of these immunological phenotypes may provide opportunities for personalised medicine in patients with AIH in the future. For patients with high BAFF, inhibitors of BAFF are a potential new treatment option in AIH. Recently, a case report was published of 2 difficult-to-treat patients with AIH who responded well to BAFF inhibition.^29^ BAFF levels were not measured in this case report. It would be interesting to know if BAFF levels were correlated with the treatment effect of BAFF inhibitors. The results of the currently ongoing phase II/III trial (NCT03217422) are eagerly awaited.

For patients with high IL-21 with a more cholestatic profile, adding UDCA could be a good option to possibly improve treatment response with few adverse effects. Screening for PSC using MRCP could be considered in these patients. A future treatment option for these patients might be low-dose IL-2, an inhibitor of IL-21. Recently, 2 patients with AIH were reported to be treated with low dose IL-2 with good clinical response in 1 patient.^30^

As AIH has different presentations, it is not surprising that different immunological phenotypes exist in patients with AIH. These could point towards different underlying immunological mechanisms that can all result in the AIH phenotype. In this study, a differentiation between 3 B-cell marker-based phenotypes with different presentation and treatment response was created, which can be used in the future for treatment differentiation: patients with high BAFF might benefit from BAFF inhibition, whereas patients with high IL-21 might benefit more from UDCA and/or low-dose IL-2 treatment. Additional studies are required to further elucidate the role of B-cell immunity and relation to treatment outcome in AIH.

## Financial support

MB was supported by unrestricted grants from Zambon Pharma, the Netherlands.

## Authors’ contributions

Methodology: MB, SH, LT, BvH. Investigation: MB, MV, CM. Formal analysis: MB. Writing – original draft: MB. Visualisation: MB. Resources: SH, RdM, AvdE, AvdM, BvH. Writing – review and editing: SH. MV, CM, RdM, AvdE, AvdM, LT, BvH. Conceptualization: LT, BvH. Supervision: LT, BvH.

## Data availability statement

Data from this study will be made available by the authors upon any reasonable request.

## Conflicts of interest

MB was supported by unrestricted grants from Zambon Pharma. No other potential conflict of interests.

Please refer to the accompanying ICMJE disclosure forms for further details.
