# Corrigendum to ‘B-cell activating factor and IL-21 levels predict treatment response in autoimmune hepatitis’ [J Hepatol (2022) doi: 10.1016/j.jhepr.2022.100460]

**DOI:** 10.1016/j.jhepr.2025.101447

**Published:** 2025-06-03

**Authors:** Maaike Biewenga, Sebastiaan Heidt, Manon Vergunst, Camiel J.M. Marijnissen, Rob A. de Man, Annemiek A. van der Eijk, Adriaan J. van der Meer, Leendert A. Trouw, Bart van Hoek

**Affiliations:** 1 Department of Gastroenterology and Hepatology, Leiden University Medical Center, Leiden, The Netherlands; 2 Department of Immunology, Leiden University Medical Center, Leiden, The Netherlands; 3 Department of Gastroenterology and Hepatology, Erasmus Medical Center, Rotterdam, The Netherlands; 4 Department of Viroscience, Erasmus Medical Center, Rotterdam, The Netherlands

It has come to our attention that the initials of Camiel J.M. Marijnissen were reversed in the published version of this article. This has now been corrected online.

