# Supplementary material for: B-cell activating factor and IL-21 levels predict treatment response in autoimmune hepatitis
Source: JHEP Rep. 2022 Feb 22;4(5):100460. doi: 10.1016/j.jhepr.2022.100460 (PMC8971938; doi:10.1016/j.jhepr.2022.100460)

B-cell activating factor and IL-21 levels predict treatment response in autoimmune hepatitis

Maaike Biewenga, Sebastiaan Heidt, Manon Vergunst, Camiel M.J. Marijnissen, Rob A. de Man, Annemiek A. van der Eijk, Adriaan J. van der Meer, Leendert A. Trouw, Bart van Hoek

Table of contents

Fig. S1 2

Fig. S2 3

Fig. S3 4

Fig. S1: gating strategy for B-cell subpopulations

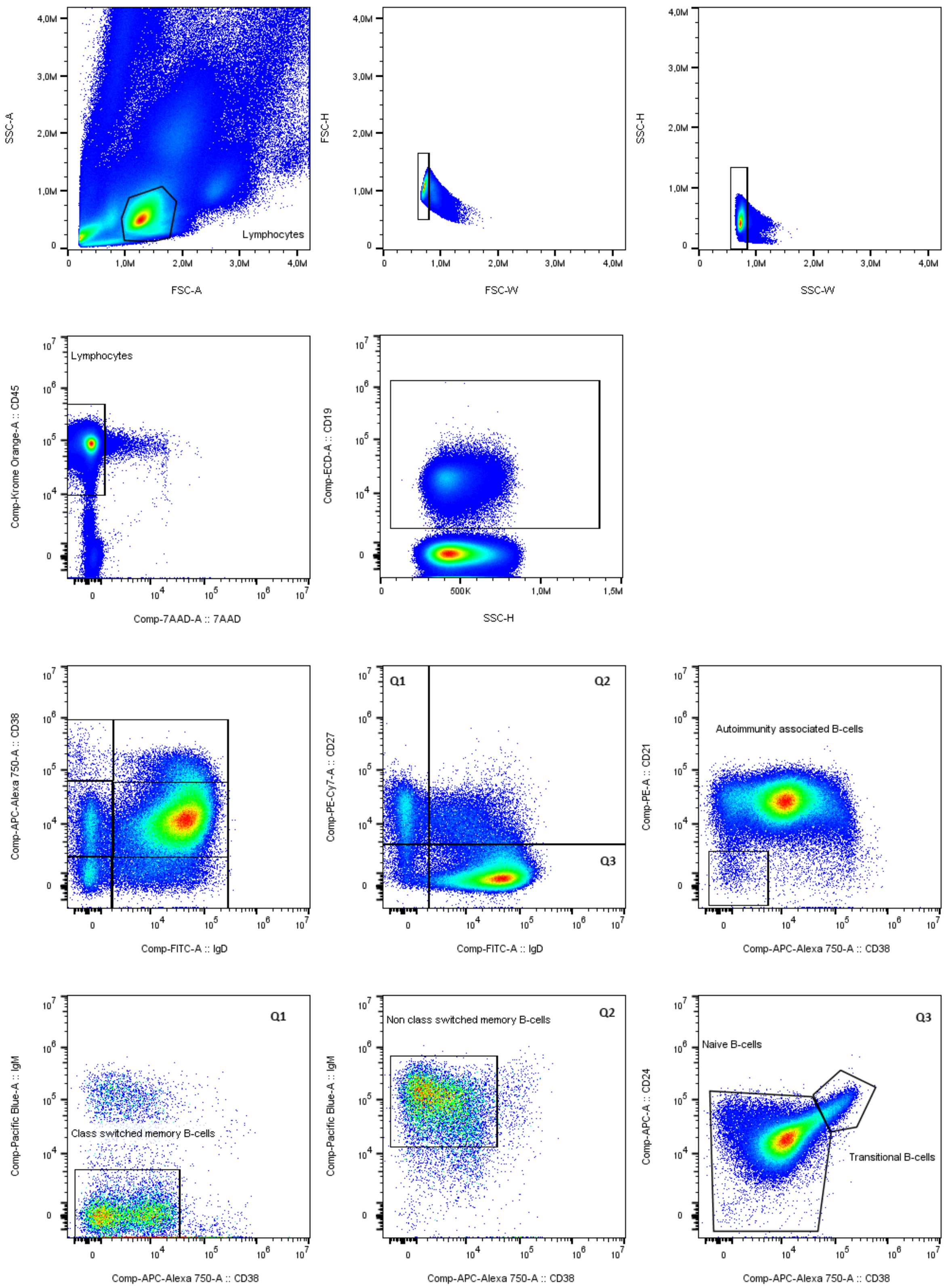

Fig. S2: correlation between BAFF, IL-21, ALT corrected for the upper limit of normal and IgG

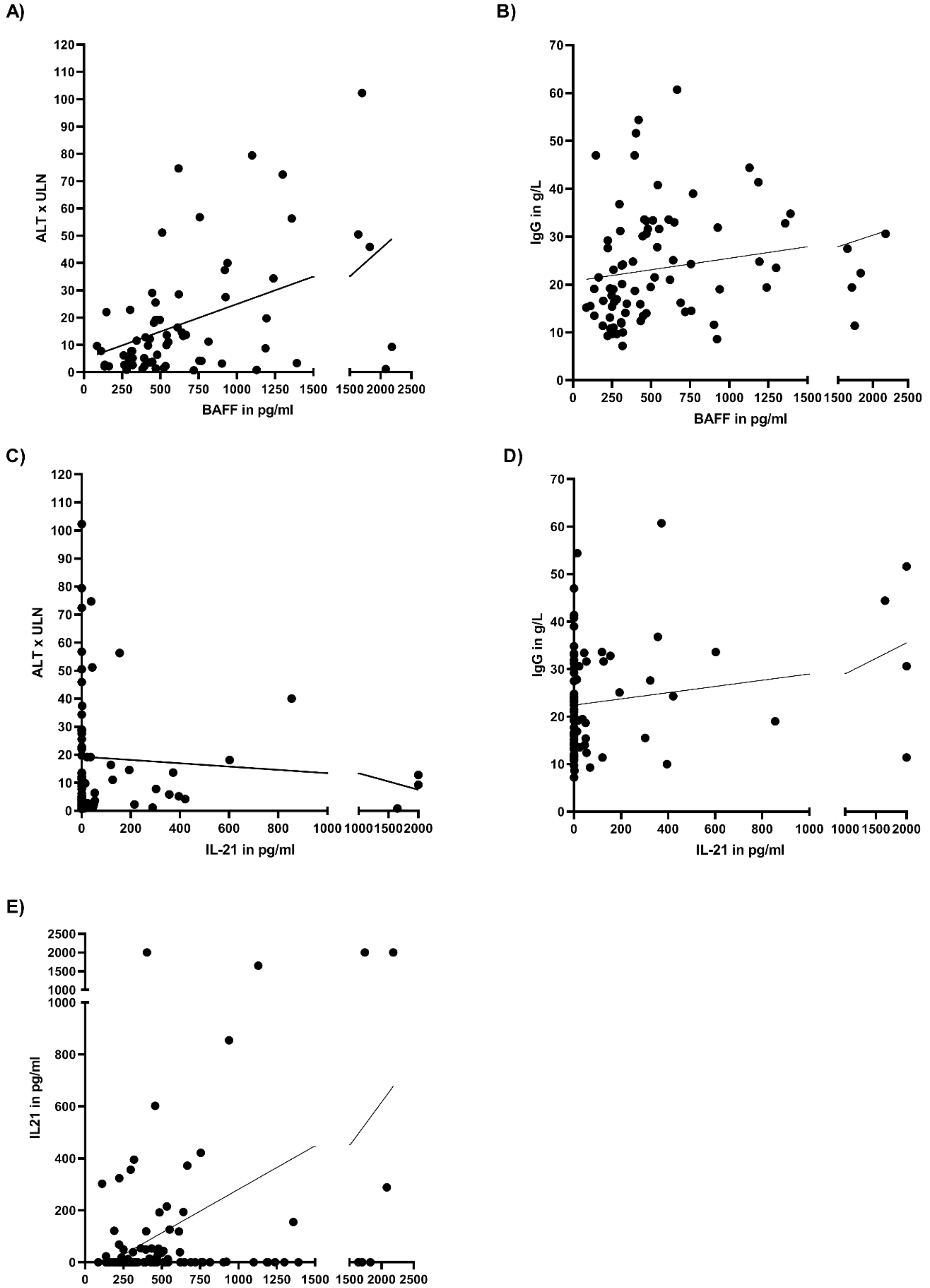

Fig. S3: Prevalence of the Bm1-5 B-cell classification in AIH patients and healthy controls. All B-cell populations are expressed as % of B-cells.

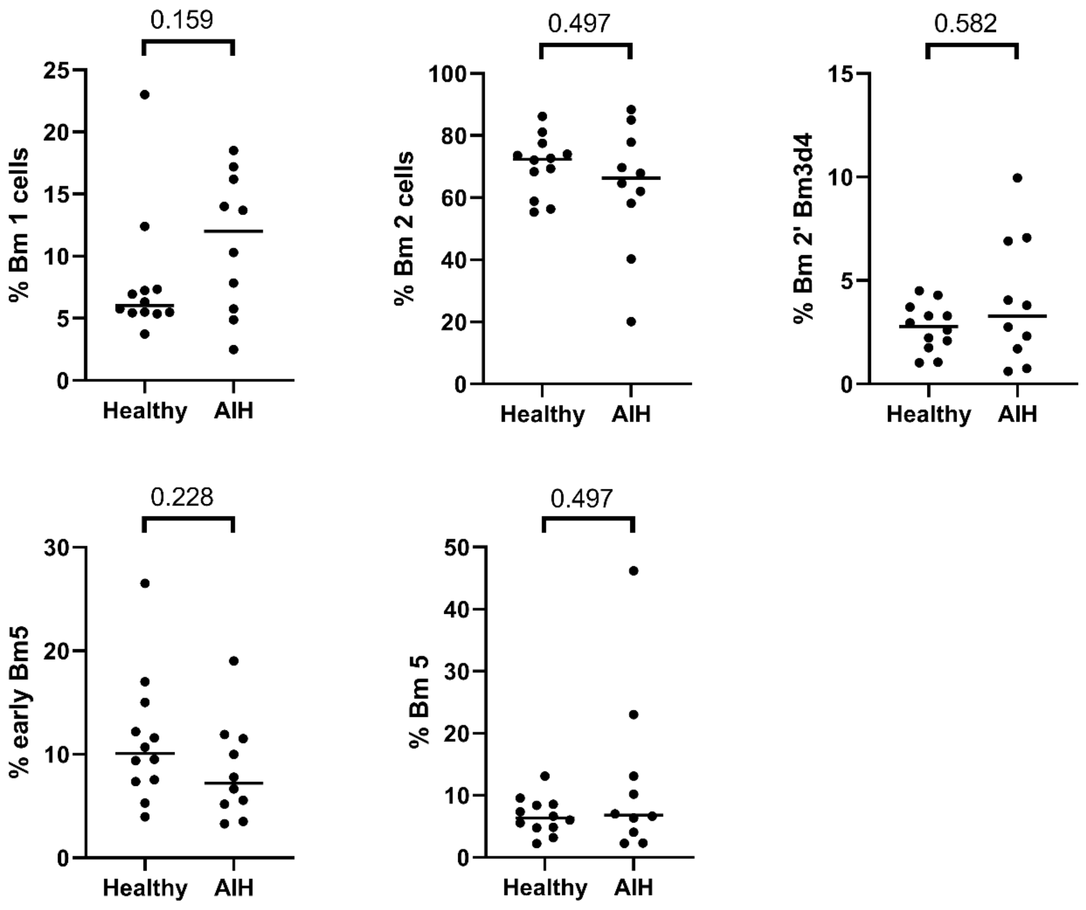

Supplement: Multimedia component 1 [file mmc1.pdf]
