# Supplementary material for: B-cell activating factor and IL-21 levels predict treatment response in autoimmune hepatitis
Source: JHEP Rep. 2022 Feb 22;4(5):100460. doi: 10.1016/j.jhepr.2022.100460 (PMC8971938; doi:10.1016/j.jhepr.2022.100460)
Supplement: Multimedia component 2 [file mmc2.docx]

**JHEP Reports**

**CTAT methods**

Tables for a “Complete, Transparent, Accurate and Timely account” (CTAT) are now mandatory for all revised submissions. The aim is to enhance the reproducibility of methods.

- Only include the parts relevant to your study
- Refer to the CTAT in the main text as ‘Supplementary CTAT Table’
- Do not add subheadings
- Add as many rows as needed to include all information
- Only include one item per row

**If the CTAT form is not relevant to your study, please outline the reasons why:**

|  |
| --- |

- 1. **Antibodies**

| **Name** | **Citation** | **Supplier** | **Cat no.** | **Clone no.** |
| --- | --- | --- | --- | --- |
| DuoSet ELISA BAFF & IL21  And:  CD45-KrO (J33), CD38-APC-A750 (LS198-4-3), CD19-ECD (J3-119), CD21-PE (BL13), CD24-APC (ALB9), CD27-PC7 (1A4CD27), IgD-FITC (IA6-2) and IgM-PB (SA-DA4 |  | R&D systems, Minneapolis, USA  **And:**  Beckman Coulter, Brea, USA  With:  Aurora-3L flow cytometer | DY8879-05 and DY124-05 |  |

- 1. **Cell lines**

| **Name** | **Citation** | **Supplier** | **Cat no.** | **Passage no.** | **Authentication test method** |
| --- | --- | --- | --- | --- | --- |
| **N/A** |  |  |  |  |  |

- 1. **Organisms**

| **Name** | **Citation** | **Supplier** | **Strain** | **Sex** | **Age** | **Overall n number** |
| --- | --- | --- | --- | --- | --- | --- |
| **N/A** |  |  |  |  |  |  |

- 1. **Sequence based reagents**

| **Name** | **Sequence** | **Supplier** |
| --- | --- | --- |
| **N/A** |  |  |

- 1. **Biological samples**

| **Description** | **Source** | **Identifier** |
| --- | --- | --- |
| **Biobank serum samples** | **LUMC** |  |

- 1. **Deposited data**

| **Name of repository** | **Identifier** | **Link** |
| --- | --- | --- |
|  |  |  |

- 1. **Software**

| **Software name** | **Manufacturer** | **Version** |
| --- | --- | --- |
|  |  |  |

- 1. **Other (*e.g*. drugs, proteins, vectors etc.)**

|  |  |  |
| --- | --- | --- |
|  |  |  |

- 1. **Please provide the details of the corresponding methods author for the manuscript:**

| Prof. Dr. B. van Hoek, MD, PhD, Professor of Hepatology Department of Gastroenterology and Hepatology, C4-P Leiden University Medical Center PO Box 9600, 2300 RC Leiden, the Netherlands Email: [B.van_Hoek@lumc.nl](mailto:B.van_Hoek@lumc.nl) |
| --- |

**2.0 Please confirm for randomised controlled trials all versions of the clinical protocol are included in the submission. These will be published online as supplementary information.**

| **N/A** |
| --- |
